# Influence of Spatial Scale on Structure of Soil Bacterial Communities across an Arctic Landscape

**DOI:** 10.1128/AEM.02220-20

**Published:** 2021-02-12

**Authors:** Lucie A. Malard, Muhammad Z. Anwar, Carsten S. Jacobsen, David A. Pearce

**Affiliations:** a Faculty of Health and Life Sciences, Northumbria University, Newcastle-upon-Tyne, United Kingdom; b Department of Environmental Sciences, Aarhus University, Roskilde, Denmark; Kyoto University

**Keywords:** Arctic soil, autocorrelation distance, bacterial diversity, biogeography, environmental factors, spatial scale

## Abstract

Bacterial community composition is largely influenced by environmental factors, and this applies to the Arctic region. However, little is known about the role of spatial factors in structuring such communities. In this study, we evaluated the influence of spatial scale on bacterial community structure across an Arctic landscape. Our results showed that spatial factors accounted for approximately 10% of the variation at the landscape scale, equivalent to observations across the whole Arctic region, suggesting that while the role and magnitude of other processes involved in community structure may vary, the role of dispersal may be stable globally in the region. We assessed dispersal limitation by identifying the spatial autocorrelation distance which would be required in order to obtain fully independent samples as approximately 60 m, and this may inform future sampling strategies in the region. Finally, indicator taxa with strong statistical correlations with environmental variables were identified. However, we showed that these strong taxon-environment associations may not always be reflected in the geographical distribution of these taxa.

**IMPORTANCE** The significance of this study is 3-fold. It investigated the influence of spatial scale on the soil bacterial community composition across a typical Arctic landscape and demonstrated that conclusions reached when examining the influence of specific environmental variables on bacterial community composition are dependent upon the spatial scales over which they are investigated. This study identified a dispersal limitation (spatial autocorrelation) distance of approximately 60 m, required to obtain samples with fully independent bacterial communities and, therefore, should serve to inform future sampling strategies in the region and potentially elsewhere. The work also showed that strong taxon-environment statistical associations may not be reflected in the observed landscape distribution of the indicator taxa.

## Abstract (precis)

The significance of this study is 3-fold. It investigated the influence of spatial scale on the soil bacterial community composition across a typical Arctic landscape and demonstrated that conclusions reached when examining the influence of specific environmental variables on bacterial community composition are dependent upon the spatial scales over which they are investigated.

## INTRODUCTION

Significant spatial structuring of soil microorganisms has been demonstrated at micro (micrometers to millimeters) (1), plot (centimeters to a few meters) (1), landscape (hundreds of meters) (2), regional (kilometers) (3), national (4, 5), continental (6), and global (7–9) scales. Hence, the scale of investigation is a key parameter to take into account in studies of bacterial biogeography. Martiny et al. (10) further demonstrated the importance of spatial scale for environmental factors identified as influencing community composition in temperate soils. They found key environmental drivers differed across spatial scales—ammonia-oxidizing bacterial (AOB) community composition was dependent on distance, moisture, and vegetation cover at the plot scale; however, at the regional scale, diversity was mainly influenced by water temperature, air temperature, and moisture, while nitrate concentration and air temperature were predominant at the continental scale. Finally, when all scales were considered together, overall key drivers were geographic distance, sediment moisture, air temperature, and vegetation cover. However, most biogeographical studies investigate communities at only one spatial scale (see references 4, 7, and 9 for further examples). The landscape scale (a few hundred meters to a few kilometers) is considered highly relevant for studies of bacterial distribution patterns, as it is the scale of human activities (at which agricultural practices and land management are integrated). Hence, the majority of studies at that scale investigate human-impacted landscapes (see references 2, 3, 11, 12, and 13 for further examples), with only a few studies describing Arctic communities from a few meters to 3 km (14–16).

**FIG 1:**
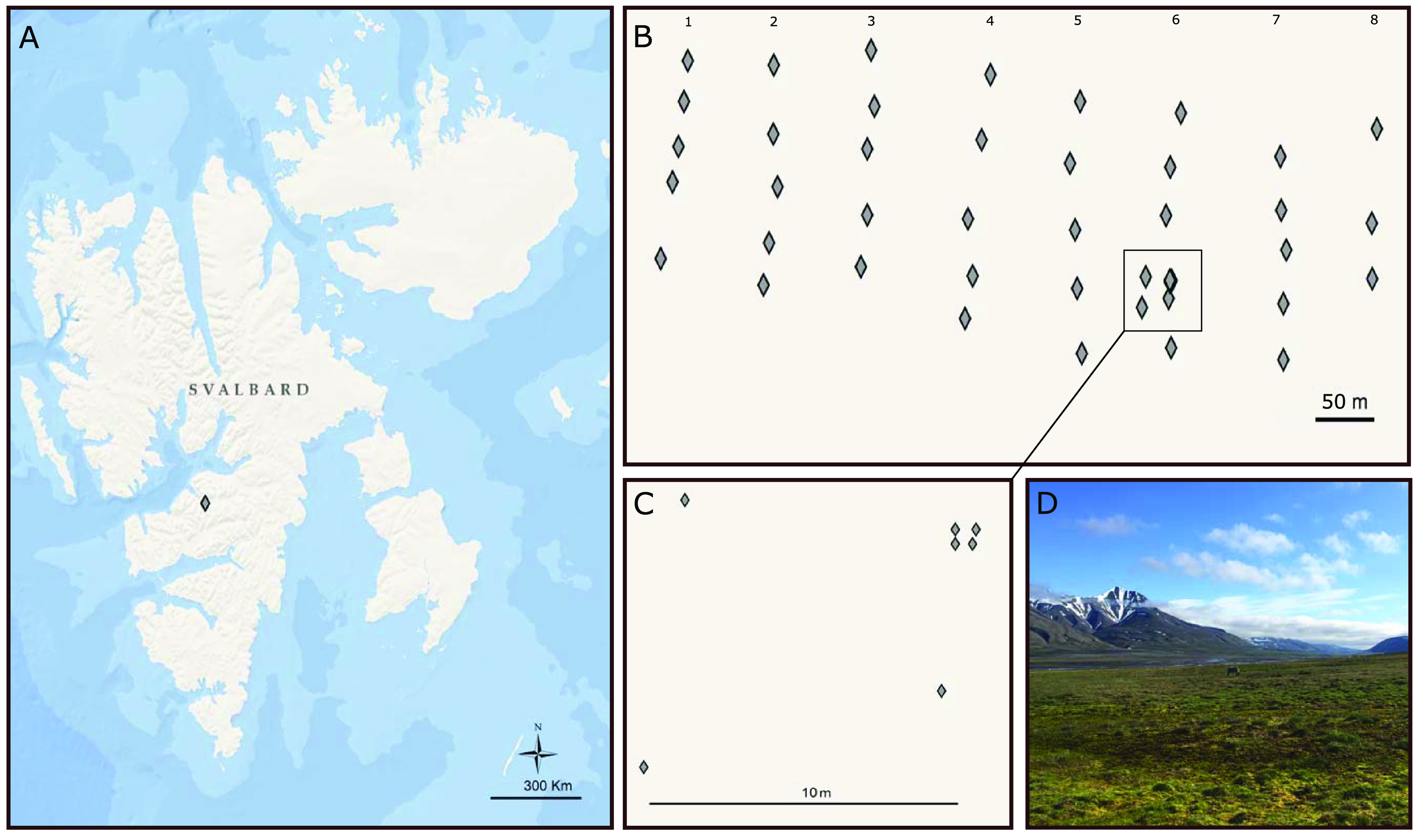
(A) Map of sampling sites in Svalbard, created using Arcmap version 10.7. (B) Sampling design in 8 transects in Adventdalen. (C) Smaller scale samples on transect 6. (D) View of Adventdalen.

The first aim of this study was to evaluate the influence of the spatial scale on bacterial community structure (see Fig. S1 in the supplemental material) across an Arctic landscape (Fig. 1). Indeed, while the role of environmental parameters such as pH (17, 18), total organic content (TOC) (19), moisture content (20), and C/N ratio (21) in community composition in the Arctic has been demonstrated, much less is known about the influence of spatial parameters (19). However, determining the influence of environmental factors on communities remains an essential step to avoid overestimating the role of the spatial scale. In addition to providing a better understanding of the environmental factors influencing community structure, investigating multiple scales provides better knowledge of the spatial structure, which facilitates the development of sampling strategies where samples are collected beyond the spatial autocorrelation distance and are, therefore, truly independent (22). As autocorrelation distances have been identified from micrometers to kilometers (22–25), with the potential of nested scales of variability (26) and site-to-site variation, no standardized protocol exists for soil sampling for metabarcoding studies (27, 28). Therefore, the second aim was to determine the minimum distance required to obtain independent soil samples in the region (Fig. S1), which may inform future sampling strategies in the Arctic. Finally, the last aim was to identify indicator taxa which were closely associated with environmental variables and map their spatial distribution across the landscape (Fig. S1). Previous studies have attempted to identify indicator taxa that could be used for environmental monitoring (for example, see references 29 and 30 [rivers] and 31 [soils]). As indicator taxa (32) highlight operational taxonomic units (OTUs) with strong environmental associations that may respond to ecological change, we expected their distribution to closely follow that of environmental parameters.

## RESULTS

### Environmental factors.

Results showed that all 35 environmental variables had a significant impact on bacterial community structure, with approximately 73% of the variance explained by environmental factors (Table 1). Overall, five key factors (TOC, pH, conductivity, aluminum levels, and arsenic levels) had the most influence on bacterial community dissimilarity, explaining 30% of variation in total. While all other environmental factors individually explained between 0.9% and 2.4% of the variation, the combined soil elemental composition (excluding pH, conductivity, and TOC) accounted for 51.5% of the total variation in bacterial community composition.

**TABLE 1:** Relative influence of environmental factors on bacterial community structure^*a*^

Variable	*R*^2^	*P*r(>*F*)
TOC	0.089	0.001***
pH	0.070	0.001***
Cond	0.059	0.001***
Al	0.041	0.001***
As	0.041	0.001***
Br	0.024	0.001***
La	0.022	0.001***
Y	0.021	0.002**
Ca	0.018	0.003**
Cl	0.018	0.001***
Cs	0.018	0.001***
Pb	0.018	0.001***
Sr	0.018	0.002**
S	0.016	0.001***
Cu	0.015	0.001***
Te	0.015	0.002**
Ba	0.014	0.003**
In	0.014	0.002**
Nb	0.014	0.004**
Nd	0.014	0.008**
Si	0.014	0.004**
Fe	0.013	0.002**
I	0.013	0.006**
Mn	0.013	0.009**
Th	0.013	0.005**
Ag	0.012	0.007**
Mo	0.012	0.013*
Sb	0.012	0.010**
Cd	0.011	0.023*
Ta	0.011	0.016*
Tl	0.011	0.021*
Zr	0.011	0.012*
Zn	0.010	0.031*
Ge	0.009	0.046*
Sn	0.009	0.036*
Residuals	0.269	NA

a Calculated by PERMANOVA using the adonis function. *P*r(>*F*), *P* value of ANOVA (output via R); NA, not applicable. *, 0.05 > *P* > 0.01; **, 0.01 > *P* > 0.001; ***, *P* < 0.001.

### Variation partitioning.

**FIG 2:**
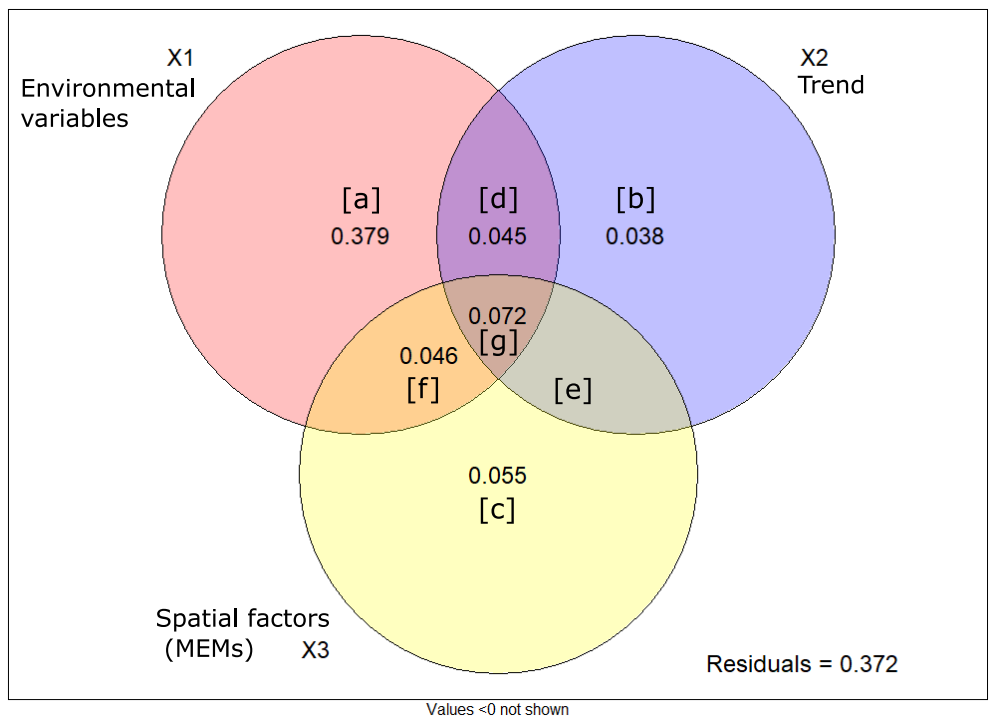
Venn diagram illustrating the results of the variation partitioning analysis on the influence of environmental variables and spatial factors on bacterial community composition. Results of each partition can be multiplied by 100 for the percentage of variation explained and are detailed in Table S2.

A total of 9 distance-based Moran’s eigenvector map (dbMEM) vectors were built using (*x*,*y*) geographic coordinates, and after forward selection, five dbMEMs were identified as significantly impacting bacterial community diversity and used in subsequent analyses. The variation partitioning analysis differentiated the effect of environmental factors, linear trend, and spatial vectors on community composition (Fig. 2). The environmental fraction X1 explained 73% of the variance (Table S1), equal to the finding by the adonis function and confirming the success of the variation partitioning analysis. Using the adjusted *R*^2^ values only as they accounted for the number of variables in the model, environmental factors explained 54% of the variance, of which 38% were not spatially structured (fraction a). The spatial component (X2 + X3) explained 25.6% of the variation, of which 16.3% could be explained by induced spatial dependence. This was illustrated by fractions d, f, and g, which represented spatially structured environmental variables where the spatial structure of these environmental variables induced a similar spatial structure in the response data, highlighting the need to evaluate the influence of the environment on communities. The remaining 9.3% of the spatial component represented spatial autocorrelation. The linear trend accounted for 3.8% of the variance (fraction b), while spatial vectors explained 5.5% of the variation. Fraction e had a negative *R*^2^ and could be considered null, as prescribed in reference 33. Each fraction (X1, X2, and X3) was tested individually and was significant (analysis of variance [ANOVA]; *P* < 0.001). In total, 62.8% of the bacterial community dissimilarity could be explained by environmental and spatial factors, while the remaining 37.2% of the variance could not be explained by the variables measured in this study.

### Spatial scale and autocorrelation.

**FIG 3:**
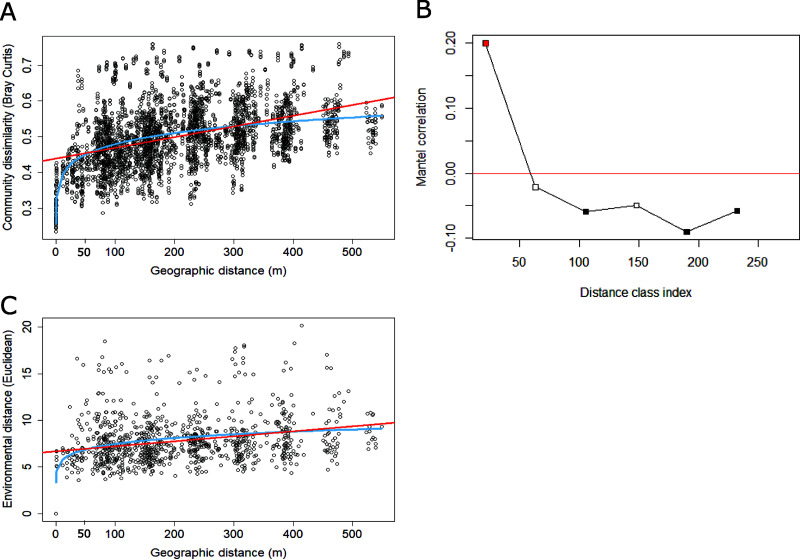
(A) Distance-decay curve illustrating the increase in bacterial community dissimilarity with increasing geographic distance. (B) Mantel correlogram of spatial autocorrelation illustrating the dispersal limitation. The red square indicates positive significant autocorrelation, which was identified only in the first distance class (0 to 21 m). Beyond 60 m, the autocorrelation was either negative (black squares) or not significant (white squares). (C) Distance-decay curve illustrating increasing environmental variation with increasing geographic distance. The red curve illustrates the linear regression, and the blue curve is the power model.

The distance-decay curve illustrated the increase in community dissimilarity with increasing distance (Fig. 3A). The power model was better fitted (*R*^2^ = 0.2261; *P* = 0.005) than the linear regression (*R*^2^ = 0.1844; *P* < 2.2 × 10^−16^). Spatial autocorrelation was visualized on the distance-decay curve (Fig. 3), where geographically close communities were more similar up to 60 m. This was illustrated with the power model on the distance-decay curve, where the blue curve begins to plateau (Fig. 3A). To further characterize the spatial autocorrelation distance, a Mantel correlogram was used (Fig. 3B) to compute the Mantel statistic between the geographic distance and bacterial community dissimilarity distance (Bray-Curtis). The spatial autocorrelation was positive for the first distance class of 21 m, indicating that the bacterial communities were more similar than expected by chance. The second distance class of 63 m displayed no spatial autocorrelation, indicating random distribution beyond 63 m. Other distance classes presented negative autocorrelations, indicating that these bacterial communities were more different than expected by chance.

Geography also had some influence on environmental conditions, with sites closer together being more similar. The spatial autocorrelation of environmental variables was first visualized in Fig. 3C, where geographically close sites were geochemically similar within 25 m. However, beyond approximately 25 m, sites equally close or far could present similar environmental conditions, as illustrated by the autocorrelation distance (Fig. 3C). This was also illustrated by the weak linear regression (*R*^2^ = 0.019; *P* < 2.2 × 10^−16^) and the best-fitted power model (*R*^2^ = 0.087; *P* = 0.005). Spatial autocorrelation was further tested for each individual variable using the semivariograms produced prior to kriging. As semivariograms are specific to each variable, the spatial autocorrelation distances were unique to each parameter. All the semivariograms produced prior to Kriging indicated positive autocorrelations oscillating between 1 m and 100 m, depending on the variable tested, further illustrating the importance of the scale of investigation (Fig. S2).

### Spatial distribution across the landscape.

**FIG 4:**
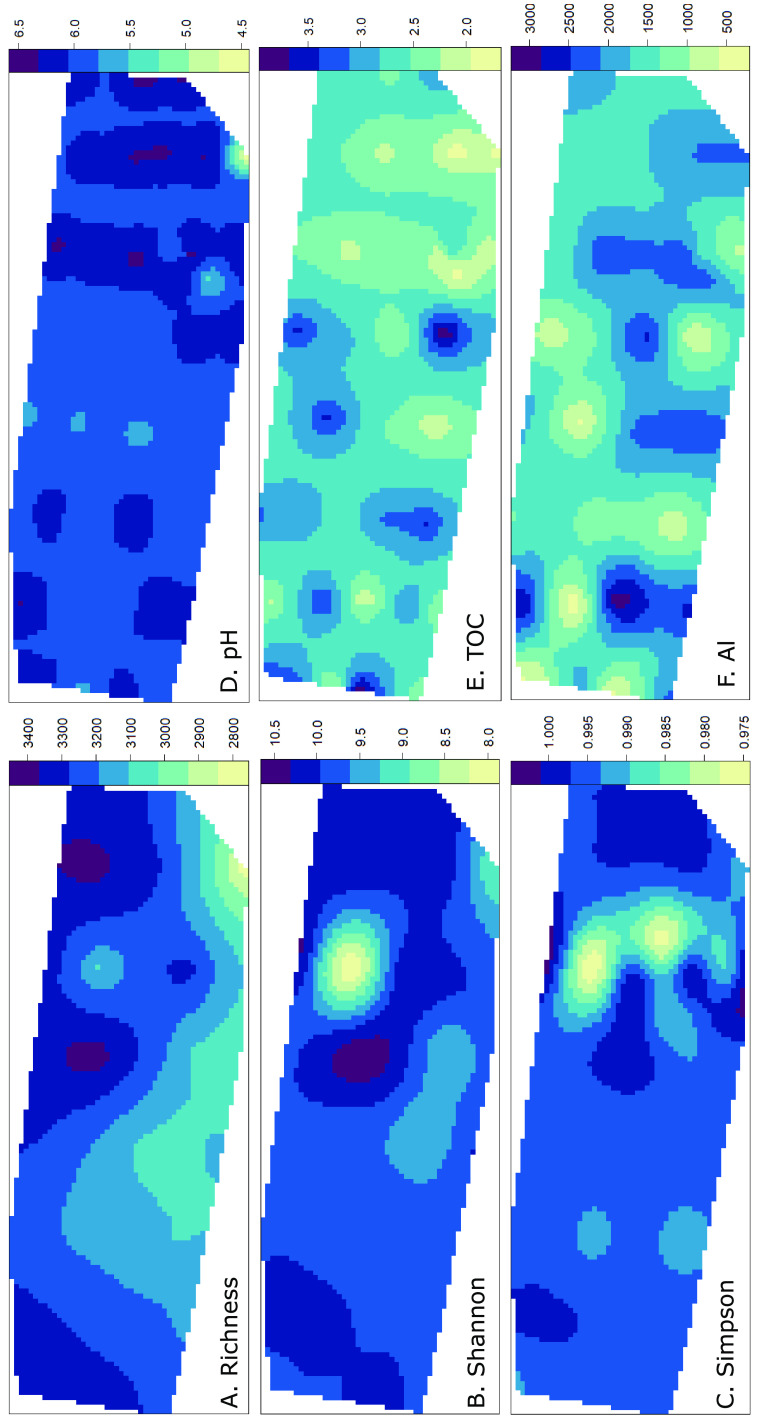
Kriged maps of the spatial distribution across the landscape showing the heterogeneity of (A) richness, (B) Shannon index, (C) Simpson index, (D) pH, (E) total organic carbon, and (F) aluminum. Thecolor bars in panels A, B, and C indicate values of alpha diversity, while those for the environmental variables indicate element concentrations (units for each variable are given in Table S2, taking into account data transformations).

Using an ordinary kriging method and after examining the semivariograms, the spatial distribution of alpha diversity and key environmental variables was mapped across the landscape (Fig. 4). The bacterial richness, diversity, and evenness changed across the landscape (Fig. 4A to C), and kriged maps illustrated the relationships between diversity, evenness, and richness. Overall, low richness indicated low diversity and low evenness, further observed using linear models (Fig. S3). The kriged maps of alpha diversity and environmental variables showed the strong heterogeneity at the landscape scale, with changes from high to low concentrations within just a few meters (Fig. 4D to F).

### Indicator taxa.

**FIG 5:**
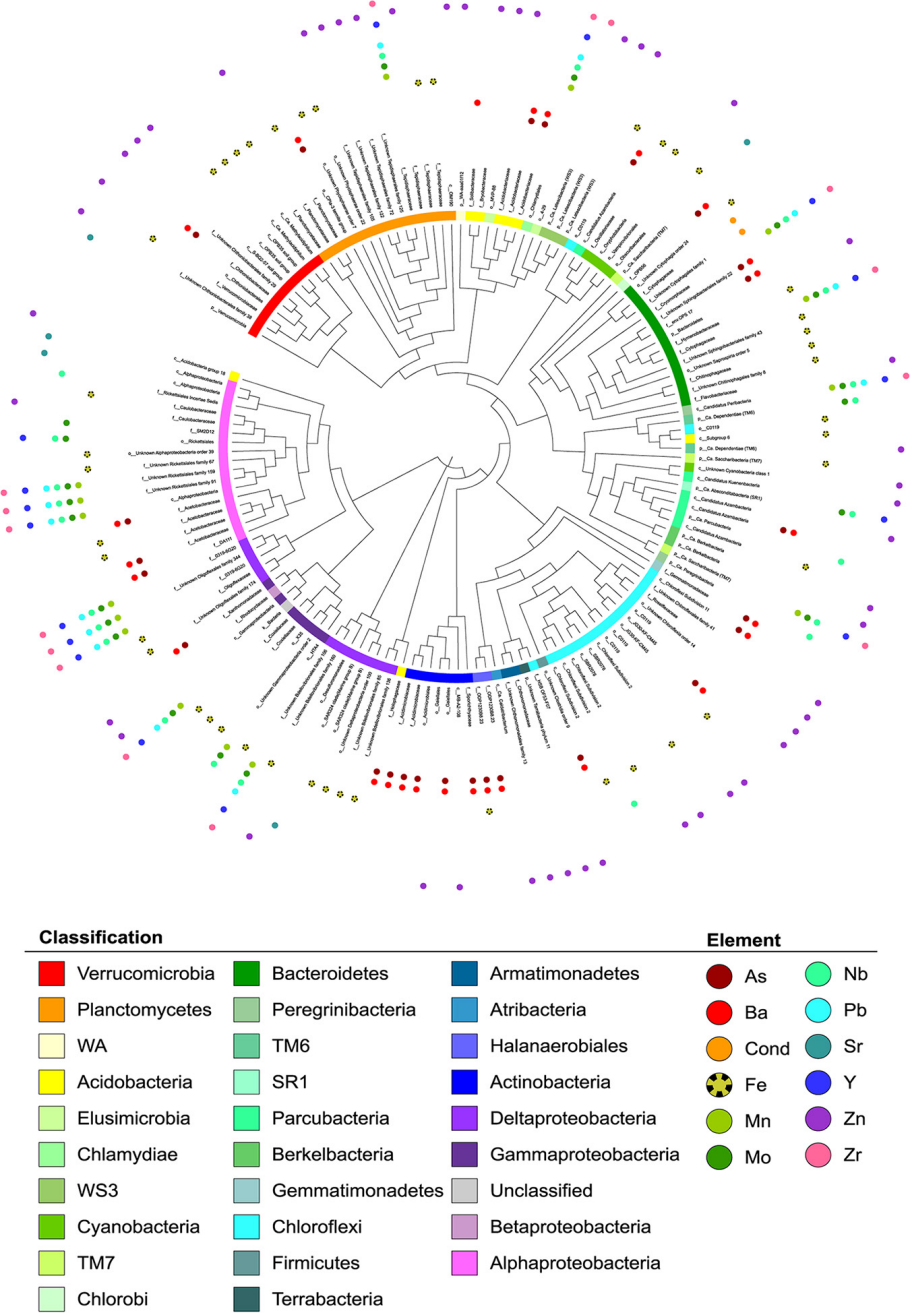
Phylogenetic tree of indicator taxa associated with environmental variables showing the high phylogenetic diversity. Colored bands illustrate the taxonomy of each OTU at the phylum level (or class level for *Proteobacteria*); labels indicate the taxonomy down to the family level if available. Colored points indicate the associated element.

**FIG 6:**
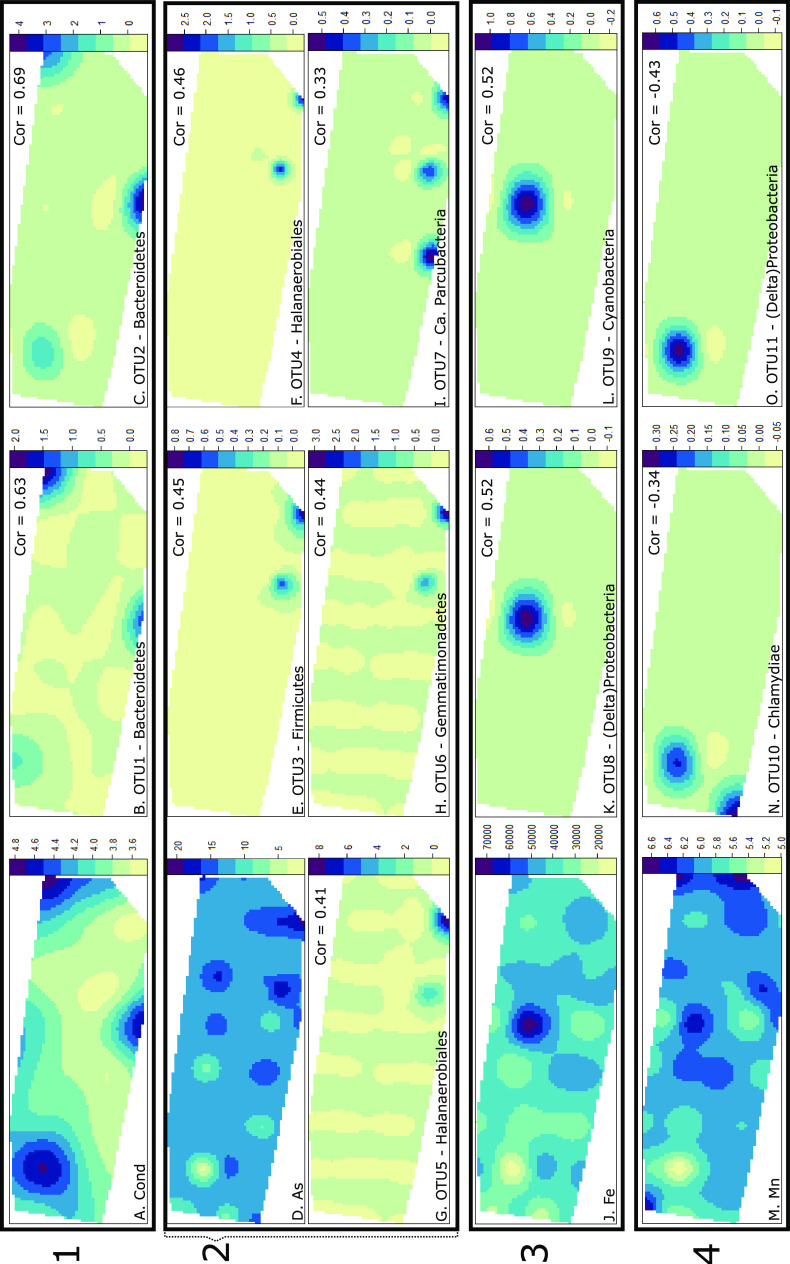
Spatial distribution across the landscape using a kriged map and illustrating the heterogeneous distribution. The color bars for environmental variables indicate element concentrations (Table S2 gives units, considering datatransformations), while those for OTUs represent relative abundance. (Box 1) (A) Conductivity. (B) Phylum, *Bacteroidetes*; order, *Cytophagales*. (C) Phylum, *Bacteroidetes*; order, *Cytophagales*. (Box 2) (D) Arsenic. (E) Phylum, *Firmicutes*; order, unknown *Clostridiales*. (F) Phylum, *Halanaerobiales incertae sedis*; order, *Halanaerobiales*. (G) Phylum, *Halanaerobiales incertae sedis*; order, *Halanaerobiales*. (H) Phylum, *Gemmatimonadetes*; order, *Gemmatimonadales*. (I) Phylum, “*Candidatus* Parcubacteria”; class, “*Candidatus* Azambacteria.” (Box 3) (J) Iron. (K) Phylum, *Proteobacteria* (*Deltaproteobacteria*); order, *Bdellovibrionales*. (L) Phylum, *Cyanobacteria*; order, *Oscillatoriales*. (Box 4) (M) Manganese. (N) Phylum, *Chlamydiae*; order, *Chlamydiales*. (O) Phylum, *Proteobacteria* (*Deltaproteobacteria*); order, *Oligoflexales*.

The indicator species analysis identified 163 true specialist (statistic >0.98) OTUs associated with 12 environmental variables. Indicator taxa were generally associated with the highest concentration of each element. The phylogenetic tree specific to indicator taxa illustrated the high taxonomic diversity of indicator taxa (Fig. 5); however, Fig. 6 demonstrates that identified indicator taxa do not necessarily follow environmental gradients as they are expected to. Of the four key factors (excluding pH) influencing bacterial communities (Table 1), only conductivity and arsenic had some associated indicator taxa. Indicators of conductivity (Cond) were restricted to two OTUs associated with high conductivity, both members of the *Bacteroidetes* classified in the order *Cytophagales* (Fig. 5). Peaks of high conductivity were visualized in Fig. 6A and correlated with peaks in abundance of the two OTUs identified (Fig. 6B and C). Indicators of arsenic (As) were closely associated with barium (Ba) and were taxonomically diverse, with the majority classified as *Actinobacteria*, *Alphaproteobacteria*, *Chloroflexi*, *Halanaerobiales incertae sedis*, and *Firmicutes* (Fig. 5). Arsenic concentration appeared more homogeneous across the landscape (Fig. 6D), with an average concentration of 13 parts per million (ppm), a minimum of 1.81 ppm, and a maximum of 20.51 ppm. These indicator taxa of arsenic were all associated with high concentrations (Fig. 6E to I) and were also associated with high concentrations of barium in the soil.

Iron (Fe) and manganese (Mn) are both essential elements of soils. Iron concentration was highly heterogeneous across the landscape, with a strong peak in concentration at one site (Fig. 6J). This peak was reflected by the presence of unique indicator taxa whose abundance was closely related to this high concentration (Fig. 6K and L). Indicators of iron were diverse, with a large number of *Proteobacteria* (*Alphaproteobacteria*, *Betaproteobacteria*, and *Gammaproteobacteria*), *Chloroflexi*, *Bacteroidetes*, *Cyanobacteria*, *Planctomycetes*, and *Verrucomicrobia* (Fig. 5). On the other hand, manganese concentration was heterogeneous across the landscape (Fig. 6M), but unlike other indicator taxa, indicators of manganese were associated with low concentrations in the soil (Fig. 6N and O). The indicator taxa of manganese were predominantly classified as *Proteobacteria* (Fig. 5) and were also closely related to low concentrations of niobium (Nb), lead (Pb), and zirconium (Zr); however, they were associated with high concentrations of molybdenum (Mo). Indicator taxa of strontium (Sr) were limited to five OTUs, an unknown *Verrucomicrobia*, a “*Candidatus* Saccharibacteria” (TM7), a *Deltaproteobacteria*, and two *Alphaproteobacteria*, while indicators of zinc (Zn) were classified in all almost all phyla (Fig. 5), illustrating the wide array of specialist taxa associated with high concentrations of zinc.

## DISCUSSION

### Key environmental factors influencing bacterial communities.

Total organic carbon, pH, and conductivity were identified as the key drivers of bacterial diversity across the Arctic landscape and are also commonly identified in studies across the globe (8, 34–38). While pH was previously identified as the primary driver of bacterial diversity in Arctic soils across the whole Arctic region (19), here, at the landscape scale, TOC was identified as the primary factor influencing bacterial community structure and was tightly linked with soil moisture. Generally, soil organic carbon content increases with increasing precipitation and decreasing temperature (39). In the Arctic tundra, not only precipitation but also snowmelt and permafrost thaw have major impacts on soil moisture and hydrology across the landscape (40, 41). In this study, where pH was on average acidoneutral at 6.05 ± 0.36 with very few acidic patches, but organic carbon content was very patchy (6% to 46%), the role of TOC in bacterial community structure is perhaps not surprising. However, it highlights the importance of investigating different spatial scales, as drivers at the global scale may not necessarily be the same across the landscape of interest.

Aluminum and arsenic were the fourth and fifth environmental variables accounting for the most variation in bacterial community structure (Table 1). Aluminum is one of the most abundant metals in the Earth’s crust, and microorganisms continuously interact with aluminum in soils (42, 43). While aluminum lacks apparent biological function (42), the aluminum ion (Al^3+^) can be toxic to living organisms and is a function of the soil pH; the concentration of toxic Al^3+^ gradually increases as pH decreases from 6.2 (42, 43). Here, small pH changes but large aluminum concentration variations were observed across the landscape, which were not correlated with each other (linear regression: *R*^2^ = 0.00069; *P* = 0.81). The toxicity of Al^3+^ may influence the bacterial community structure; however, the concentration of Al^3+^ ions was not measured.

Arsenic is ubiquitous in low abundance in the natural environment and recognized as one of the most toxic elements (44, 45). Here, a decrease in diversity and richness was observed with increasing arsenic concentrations, which likely reflects the toxic effect of oxyanions of arsenate on many bacteria, although some can use it as a terminal electron acceptor (44). As with Al^3+^, the chemical concentration of the various forms of arsenic was not measured, and therefore, we cannot conclude that the toxicity has an influence on bacterial community structure, although it is a possibility. Indicator taxa associated with high concentrations of arsenic were diverse but dominated by *Actinobacteria* and *Proteobacteria*, which was in accordance with results reported by Dunivin et al. (45), who conducted a global survey of arsenic related genes in soils and identified these phyla as harboring more arsenic resistance genes.

All other elements measured had some influence on the observed bacterial community (Table 1), from key major elements such as sulfur, calcium, and silicon to key trace elements such as iron, manganese, magnesium, zinc, copper, molybdenum, and cadmium, as well as other elements, toxic or not, such as bromine, yttrium, and lead. It should also be noted that while TOC, pH, and conductivity had a significant influence on bacterial community composition (21.8%), the soil elemental composition combined explained most of the variation (>50%). This may serve to highlight the level of complexity of the factors influencing community structure.

### Indicator taxa.

Environmental variables were highly heterogenous across the landscape, which was reflected by the distribution of alpha diversity and indicator taxa. The indicator species analysis determined abundant OTU-environment associations and identified 163 OTUs that could be considered true specialists in relation to 12 environmental variables. These OTUs were generally associated with high concentrations of the variable in question except for those associated with manganese, niobium, lead, and zirconium, which were representative of low concentrations. As illustrated in the phylogenetic tree (Fig. 5), the diversity of these indicator taxa was high, with numerous representatives of the *Proteobacteria*, *Chloroflexi*, *Bacteroidetes*, *Planctomycetes*, and *Verrucomicrobia*. The distribution of some indicator taxa, selected for their reported relationship with the associated variable in the literature, was mapped across the landscape to illustrate the association with the elements’ concentrations. For arsenic, *Clostridium* and *Clostridia*-related (*Halanaerobiales*) taxa were mapped, as they have been identified with some role in arsenic cycling (44, 46) and with arsenic resistance genes (45). A *Gemmatimonadetes* and a “*Candidatus* Parcubacteria” (clustered closely with the *Cyanobacteria*) were also mapped, as both have been identified with potential roles in arsenic cycling (46). The distribution of OTUs associated with iron were mapped and included a *Cyanobacteria* (47, 48) and a *Deltaproteobacteria*, a class with known taxa involved in iron cycling (47–49). Finally, the OTUs associated with manganese were also associated with other environmental variables and mainly identified as *Proteobacteria*. A *Deltaproteobacteria* and the only *Chlamydiae* identified were mapped, two classes associated with manganese cycling (48). While this analysis showed the strong associations of some OTUs with the measured environmental parameters, it also illustrated the difficulty of using indicator taxa for monitoring purposes due to the large number of associations identified and the high heterogeneity across the landscape. This was clear when the distribution of key indicator taxa was mapped across the landscape and did not clearly follow the distribution of the environmental variable associated. Furthermore, while indicator taxa may be identified, they do not necessarily participate in the associated element cycle. For instance, these OTUs may benefit from high concentration of arsenic due to higher tolerance to toxicity and decreased competition, without having any involvement in arsenic cycling.

### Selection and dispersal structure bacterial communities.

The variation partitioning analysis quantified the importance of both selection (deterministic) and dispersal (stochastic) for bacterial community structure. Environmental variables explained 54% of the total variation, corresponding to selection, and 16% were spatially structured, corresponding to the induced spatial dependence. Then, spatial components (trend + dbMEMs) alone explained 10% of the variation, illustrating spatial autocorrelation or dispersal (33). This is the magnitude of influence recorded by Malard et al. (19), who investigated biogeographical patterns across the whole Arctic region, suggesting that the magnitude of influence of dispersal of bacterial community structure may be stable in the Arctic.

More specifically, the distance-decay curve of environmental factors showed that edaphic properties were spatially autocorrelated up to approximately 25 m, although this was the overall spatial autocorrelation, as each variable autocorrelated within different distances. After that distance, environmental variables were independent, and this was illustrated by the weak slope of the linear regression and the overall variability of edaphic properties. In addition, even highly similar environmental conditions could harbor dissimilar bacterial communities, further illustrating the potential role of dispersal and other processes, such as drift or diversification. The distance-decay curve of bacterial communities showed a positive spatial autocorrelation distance at up to 60 m, which was further supported by the Mantel correlogram. For the Arctic region as a whole, an autocorrelation distance within the same order of magnitude, approximating 20 m, was previously identified (19). This limited dispersal range in Arctic soils is in contrast with studies in other regions of the globe. For instance, in a glacier forefield in southern Alaska, this distance was over 600 m (50) while in British soils, it was below 1 km (4). This suggests that Arctic soil bacterial communities disperse only to approximately 60 m and may form rather isolated island communities. Thus, the scale of sampling is important in these landscapes to capture community variability, and therefore, a minimum of 60 m should be maintained between sites to obtain independent samples. Further investigations at other Arctic sites are required to determine whether this applies across the whole Arctic region.

Overall, these results suggest that induced spatial dependence may be an important factor shaping bacterial communities within 25 m; that is, as edaphic properties are very similar, bacterial communities are also similar. Between 25 and 60 m, environmental variability increased, and yet communities remained relatively similar, suggesting that dispersal may be the primary process shaping bacterial communities. Beyond 60 m, the environment was highly heterogeneous, bacterial communities were highly dissimilar, and selection was likely the main process structuring communities. While one process may dominate within each distance category, it is still likely the combination of different processes (selection, dispersal, diversification, and drift) with different magnitudes still driving community assembly (51).

While 63% of the variation (nonadjusted *R*^2^ = 81%) of bacterial community assemblage could be explained, 37% remained unexplained. Many factors, whether biotic or abiotic, could influence bacterial communities. Based on the scale of this study, it is unlikely that most climatic and topographic variables would have much influence on the community structure variation. Instead, other edaphic factors, such as total nitrogen or phosphorus content and clay, silt, and sand content, but also the presence of ice or soil texture may have more impact locally. Furthermore, biotic interactions such as competition and predation within bacterial communities or with other members of the soil biota or higher organisms may have some influence. For instance, grazing is one of the main disturbances to the ecosystem locally, primarily by Svalbard reindeer and the barnacle goose (52). In addition to impacting the vegetation, they trample over the landscape and fertilize it; therefore, grazing can have significant impacts on the ecosystem, and it has been shown to decrease microbial respiration and the available carbon (53), while animal feces increase the available nitrogen and can increase bacterial abundance (54). Human presence may also have some influence, as the sampling site was close to another scientific research site with open-top chambers. A few cabins were located in the area, and coal mine 7 was still in operation, approximately 1.5 km away and 400 m above the sampling site.

### Conclusion.

In this study, spatial factors accounted for approximately 10% of the variation in community composition at the landscape scale, equivalent to observations across the whole Arctic region, suggesting that while the role and magnitude of other processes involved in community structure may vary, the role of dispersal may be stable globally in the region. Furthermore, the identification of different driving environmental factors at different scales highlights their dependence upon the spatial scales over which they are investigated. Overall, we suggest that induced spatial dependence may shape bacterial communities within 25 m. Between 25 and 60 m, dispersal may be the primary process shaping bacterial communities, and beyond 60 m, selection is likely the main process structuring communities. As dispersal may be limited to 60 m, and while further studies should be conducted, we suggest that soil sampling in the region should be conducted beyond this distance to capture landscape variability when independent samples are being collected. Finally, by mapping the spatial distribution of indicator taxa across the landscape, we showed that strong taxon-environment statistical associations may not actually be reflected in the landscape distribution of these bacterial taxa.

## MATERIALS AND METHODS

### Sampling site.

In July 2017, 44 soil samples were collected in Adventdalen, Svalbard, Norway (Fig. 1A), following the sampling design depicted in Fig. 1B and characterized by 8 north-south transects of 5 samples each. Samples within each transect were approximately 50 m apart, while the distance between transects was approximately 100 m. On transect 6, extra samples were collected 10 m and 1 m apart to investigate smaller-scale patterns (Fig. 1B and C).

Adventdalen is a broad U-shaped valley open to the west, from which the mouth is located approximately 2 km from Longyearbyen and 6 km from Svalbard Airport. Adventdalen was deglaciated about 10 kiloannums before the present (ka BP) (55), and permafrost is estimated to be 100 m thick close to the shore. It is a typical Arctic landscape, in one of the driest areas of Svalbard, with an average of 190 mm of annual precipitation and a mean annual temperature of −6°C (56). The study site was located approximately 9 km into the valley, 11 km away from Longyearbyen, at 78.17°N, 16.02°E. The vegetation is primarily dwarf shrub/grass heath, dominated by *Salix* spp., mosses, lichens, and *Graminea* spp. (57) (Fig. 1D). The main disturbances to the site come from grazing, primarily by Svalbard reindeer (*Rangifer tarandus platyrhynchus*) and the barnacle goose (*Branta leucopsis*) (52).

### Sample collection and soil properties.

The coordinates of each site were recorded with a portable global positioning system (GPS) device. At each location, 50 g of soil in the top 15 cm was collected using ethanol-cleaned trowels and Whirl-Pak bags (Nasco, Fort Atkinson, WI, USA). Plant roots and rocks were removed manually in a class II biological safety cabinet (ESCO, Singapore); samples were homogenized and frozen at −20°C before transportation to the United Kingdom for analyses. pH and conductivity were measured in the laboratory at a 1:5 ratio of freshly thawed soil to water, using a Mettler-Toledo FE20 pH meter (Mettler-Toledo Instruments Co., Shanghai, China) and a CMD500 conductivity meter (WPA, Cambridge, UK). Moisture content was measured gravimetrically on soils after drying at 150°C for 24 h, and total organic content (TOC) was measured gravimetrically by heating previously dried soils to 550°C for 4 h. To analyze the elemental composition of each sample, 5 g of thawed soil was placed in ceramic crucibles and left to dry at 37°C for 5 days. Dried samples were crushed to a fine powder using a mortar and pestle, put in a powder sample cup, placed in the XRF spectrometer (X-Lab2000; Spectro, Kleve, Germany) and analyzed. Resulting concentrations were adjusted using calibrated values and results were expressed in parts per million.

### DNA extraction and amplicon sequencing.

Soil DNA was extracted in duplicate for each sample using the PowerSoil kit (Qiagen, Hilden, Germany) following the manufacturers’ protocol. 16S rRNA gene libraries were constructed using the universal primers 515F and 806R (58) to amplify the V4 region. Amplicons were generated using a high-fidelity Accuprime DNA polymerase (Invitrogen, Carlsbad, CA, USA), purified using AMPure magnetic bead capture kit (Agencourt, Beckman Coulter, MA, USA), and quantified using a QuantIT PicoGreen fluorometric kit (Invitrogen). The purified amplicons were then pooled in equimolar concentrations using a SequalPrep plate normalization kit (Invitrogen), and the final concentration of the library was determined using a SYBR green quantitative PCR (qPCR) assay. Libraries were mixed with Illumina-generated PhiX control libraries and our own genomic libraries and denatured using fresh NaOH. The resulting amplicons were sequenced on the Illumina MiSeq V2 (500 cycles).

### Bioinformatics processing.

Raw paired-end reads were subjected to adaptor and primer clipping using Cutadapt (59), resulting in 71,207 ± 3,280 reads per sample. Forward and reverse reads were merged using FLASH (60). Reads with more than 1.5 total expected errors per read and/or read lengths less than 245 bp were truncated during quality filtration using the Vsearch (61) filtering module. This resulted in 64,917 ± 4,291 high-quality merged reads per sample. Dereplication was performed to identify unique sequences. A two-step chimera detection method was used, first by aligning against ChimeraSlayer Gold database provided with SILVA (62) and second by using the *denovo* detection module. An open-reference operational taxonomic unit (OTU) calling was performed on high-quality trimmed sequences at a 97% similarity level using the USEARCH (63) algorithm for clustering to generate operational taxonomical units (OTUs). This resulted in (85 DNA samples) a total of 5,436,264 reads (63,956 ± 38,865 reads/sample) assigned against 23,627 OTUs. Unique (chimera-filtered) representative sequences were aligned using the Python Nearest Alignment Space Termination (PyNAST) (64) tool with a relaxed neighbor-joining tree built using FastTree (65). OTUs were assigned taxonomy using the lowest common ancestor (LCA)-based Classification Resources for Environmental Sequence Tags (CREST) package (66), with a minimum classification score of 0.80 against SILVA release 128 as a reference database.

### Statistical analysis.

Alpha diversity (richness and Shannon and Simpson indices) was calculated in QIIME v1.90 (67) on the matrices resulting from multiple rarefactions, with the smallest sample size (22,316 reads) as the maximum depth. Results were collated and averaged to obtain a single representative value for each sample. The OTU table was normalized using cumulative-sum scaling (CSS) (68). The resulting OTU table was input into R for subsequent analyses, and the Bray-Curtis dissimilarity distance was calculated using vegan (69).

To evaluate the environmental component, Pearson’s correlation coefficients were calculated using the corrplot package (70) to first identify possible correlations between environmental variables. With this many variables, it was a necessary step to avoid misinterpretation of the results (76). Coefficients over |0.8| indicated strong correlations (Fig. S4), and as such, variables were removed to keep only one representative (76). For example, a high moisture content was correlated with a high TOC content (Pearson’s coefficient = 0.88); in this case, moisture was discarded, as it is weather dependent and is expected to be more variable day to day than TOC. Of 48 parameters measured, 35 were independent and considered representative. The distribution of the 35 remaining environmental variables was investigated using the moments package (71) to assess the skewness and kurtosis. Skewness evaluates the degree of distribution shift to one side or another, and a good distribution should equal 0, while kurtosis evaluates the tail distribution and should also be close to 0 to assume normal distribution. Using diagnostic plots, skewness, and kurtosis, the necessary transformations to improve the unimodal distribution of environmental variables were carried out (summarized in Table S2), and collinearity was verified again with Pearson’s correlations (Fig. S5). Transformed environment variables were scaled, and a sequential permutational multivariate ANOVA (PERMANOVA) was conducted using the adonis function implemented in vegan with standard 999 permutations to identify environmental variables significantly associated with the Bray-Curtis community dissimilarity.

To evaluate the spatial component, the geographic locations (*x*,*y*) of the sampling sites were transformed to Cartesian coordinates using the SoDA package (72), and the Euclidean distance was calculated using vegan. Distance-decay curves were produced using linear regressions of the Euclidean distance of the geographic locations against the Bray-Curtis dissimilarity distance and the Euclidean distance of scaled environmental variables.

The presence of a linear trend (a systematic increase or decrease in the OTU data with (*x*,*y*) coordinates) was visualized by the distance-decay curve (Fig. 3A) and tested by redundancy analysis (RDA) and ANOVA, as prescribed by Borcard et al. (33). As a significant linear trend was identified, the OTU table was detrended by linear regression of the (*x*,*y*) coordinates. Distance-based Moran’s eigenvector maps (dbMEMs) were constructed with (*x*,*y*) coordinates using the adespatial R package (73). The significance of the spatial vectors (dbMEMs) was assessed using the detrended OTU table and tested with ANOVA. Forward selection was conducted to identify significant dbMEM vectors, and the remaining dbMEMs were plotted using RDA.

Variation partitioning analysis (VPA) was used to assess the impact of environmental and spatial factors on community composition (undetrended OTU table) and was conducted using the environmental variables, (*x*,*y*) coordinates (linear trend), and significant dbMEM vectors. Individual fractions were tested using RDA and ANOVA, as prescribed by Borcard et al. (33).

To evaluate spatial autocorrelation, the detrended OTU table and the Euclidean distances of Cartesian coordinates (*x*,*y*) were used to produce a Mantel correlogram with standard 999 permutations using vegan. Semivariograms were also produced using the autoKrige function of the automap package (77) to use for geostatistical analyses. Kriging was conducted using the autoKrig and automapPlot functions in the automap package. Environmental variables and alpha diversity measures were interpolated and mapped across the landscape.

Indicator taxa were determined by the Dufrene-Legendre indicator species analysis (32) to identify OTUs that were specifically associated with different environmental variables. The first step was to define categories for each environmental variable (i.e., high conductivity, medium conductivity, and low conductivity). To identify groups statistically rather than subjectively, an automatic cluster approach was employed using the nbclust package (74), which indicated the ideal number of groups (Table S2). Clusters were created using the kmeans function (Table S2) and used with the multipatt function in the indicspecies package with 999 permutations (32). Indicator taxa with a correlation statistic higher than 0.98 were considered true specialists and used for subsequent analyses. The phylogenetic tree of indicator taxa was built using the representative sequences from the identified indicator taxa using FastTree method (65) and visualized using iTOL (75). Indicator taxon distribution was mapped across the landscape by kriging, as previously described, and Pearson correlations were calculated between the indicator taxa and the environmental variables of interest.

### Data availability.

The sequencing data set is deposited at the European Nucleotide Archive under BioProject accession no. PRJNA564217.

## Supplementary Material

Supplemental file 1
