# Supplementary material for: Influence of Spatial Scale on Structure of Soil Bacterial Communities across an Arctic Landscape
Source: Appl Environ Microbiol. 2021 Feb 12;87(5):e02220-20. doi: 10.1128/AEM.02220-20 (PMC8090890; doi:10.1128/AEM.02220-20)
Supplement: Supplemental file 1 [file AEM.02220-20-s0001.pdf]

Supplementary Figures and Tables

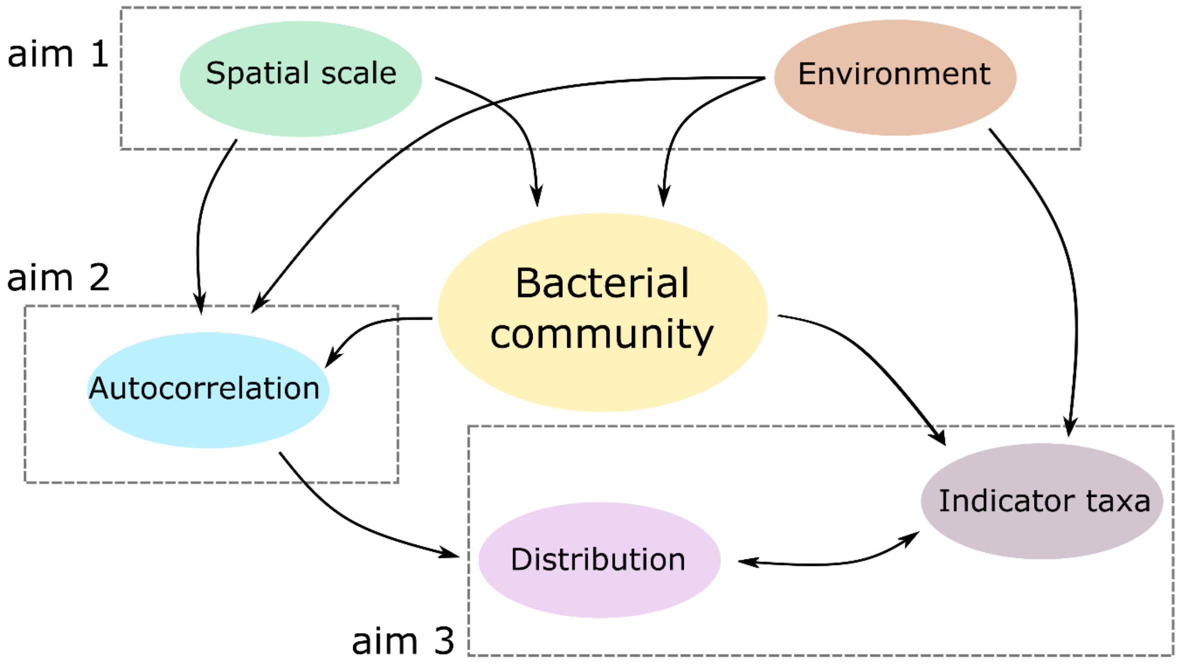

Figure S1: Diagram of the study's aims. The first aim was to evaluate the influence of the spatial scale and environmental properties on bacterial community structure. The second aim was to determine the spatial autocorrelation distance to estimate the minimum distance required to obtain independent soil samples in the region. Furthermore, determining the autocorrelation distance was essential to map spatial distribution across the landscape. The last aim was to identify indicator taxa associated with environmental variables and map their distribution across the landscape.

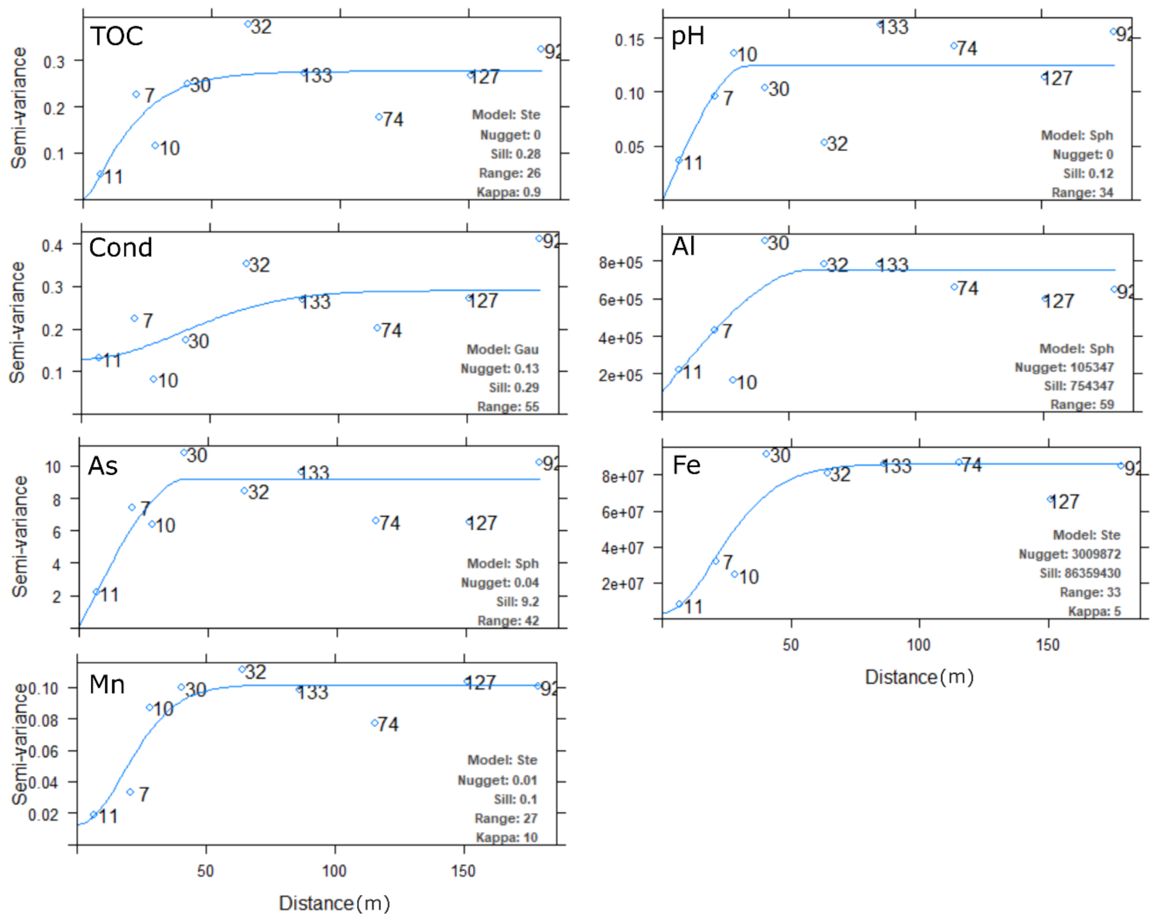

Figure S2: Semi-variograms of the variables of interest plotted with the autoKrig function which finds the best model for the data provided. Points display the number of sample pairs that went into calculating each variogram lag. These variables were selected for plotting based on the indicator taxa later identified. All other variables used in the study presented similar semi-variograms.

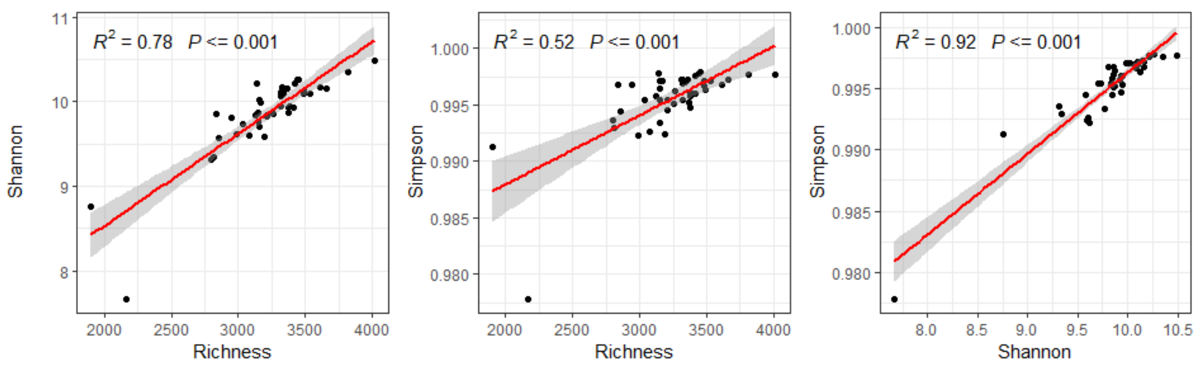

Figure S3: Linear models illustrating correlations between alpha diversity measures.

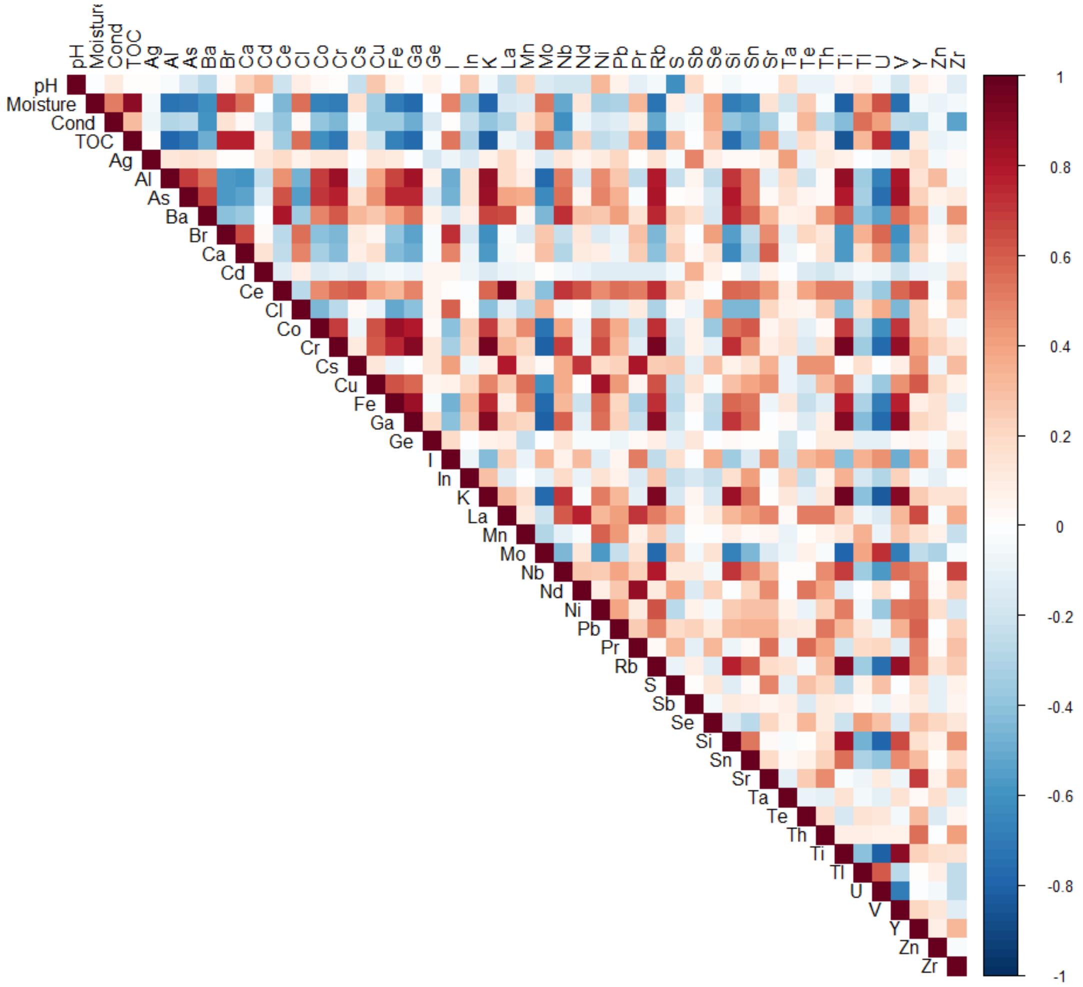

Figure S4: Pearson correlations of all measured environmental variables prior to removing collinear variables.

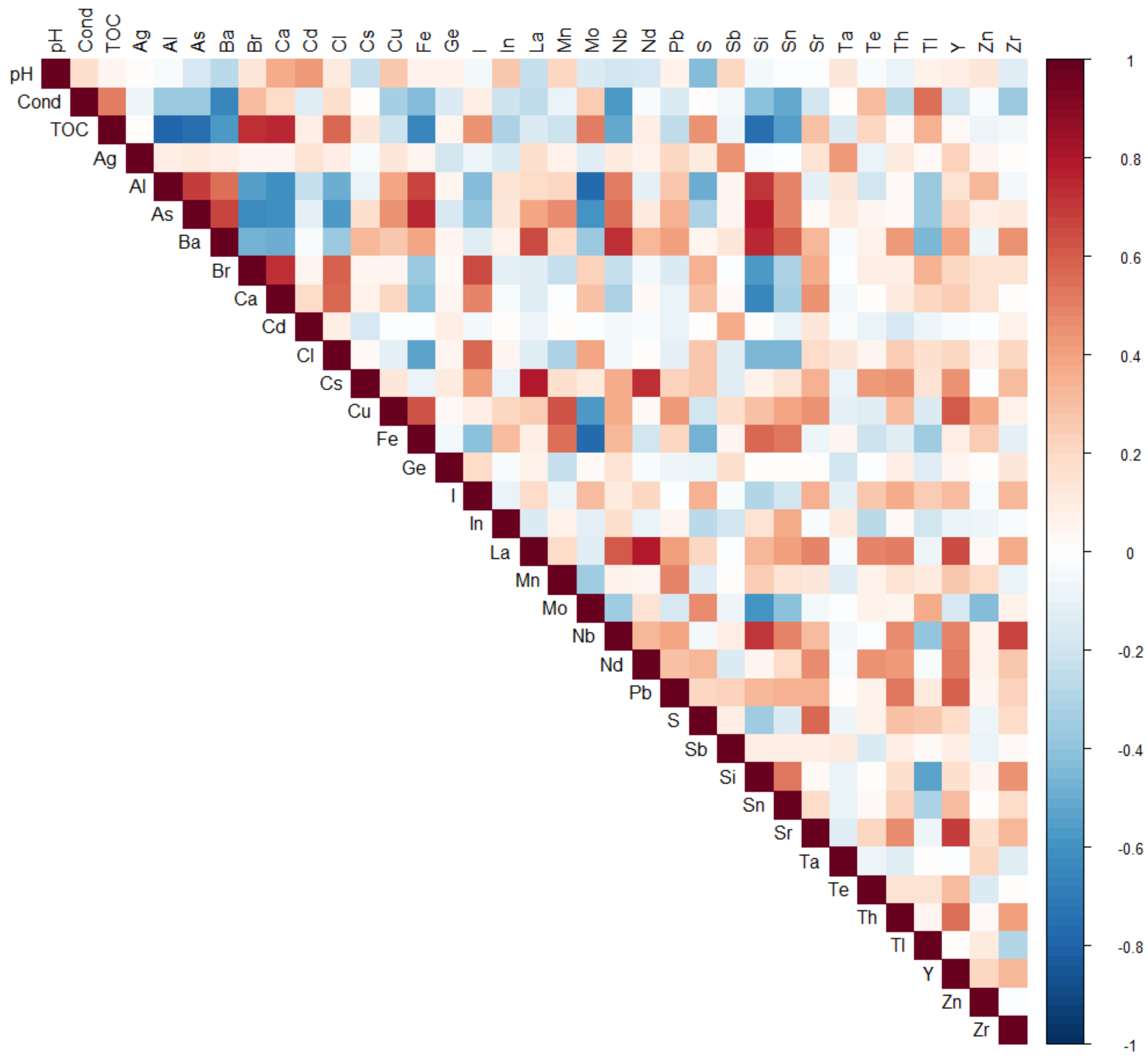

Figure S5: Pearson correlations of environmental variables used in all the analyses after removing collinear variables and transforming the remaining to attain normal distribution. All coefficients are below the |0.8| threshold.

Table S1: Results of the variation partitioning analysis. Partition X1 represents the environmental variables, partition X2 represents the linear trend while partition X3 represents the spatial vectors (dbMEMs).

Partition table	Df	R^2	Adj. R^2	Individual fractions	Df	R^2	Adj. R^2
[a+d+f+g] = X1	35	0.733	0.542	[a] = X1 | X2+X3	35	N/A	0.379
[b+d+e+g] = X2	2	0.167	0.147	[b] = X2 | X1+X3	2	N/A	0.038
[c+e+f+g] = X3	5	0.215	0.165	[c] = X3 | X1+X2	5	N/A	0.055
[a+b+d+e+f+g] = X1+X2	37	0.760	0.572	[d]	0	N/A	0.045
[a+c+d+e+f+g] = X1+X3	40	0.785	0.590	[e]	0	N/A	-0.008
[b+c+d+e+f+g] = X2+X3	7	0.311	0.249	[f]	0	N/A	0.046
[a+b+c+d+e+f+g] = All	42	0.814	0.628	[g]	0	N/A	0.072
				[h] = Residuals	N/A	N/A	0.372

Table S2: Table of the 35 environmental variables used for analyses and the transformations carried to attain normal distribution. Min, max and mean concentrations are in ppm except for pH, conductivity (mS) and TOC (%). NbClust was used to determine the best number of clusters and kmeans was used to determine the center of these clusters to use in the indicator analysis.

Variable	Min	Max	Mean ± SD	Transformation	Number of clusters	Cluster centers
pH	4.4	6.5	6.05 ± 0.36	None	3	4.4/5.8/6.3
Conductivity (mS)	23.2	227	60.74 ± 46.96	Log	3	37.7/73.2/216.7
TOC (%)	6.08	47.6	16.12 ± 10.11	Log	2	10.4/29.1
Ag	0	1	0.22 ± 0.32	Square root	2	0.02/0.6
Al	0	3382	1725 ± 858.87	None	4	385/11887/1712/2500
As	1.8	20.5	13.13 ± 3.38	None	5	7.4/12.7/14.8/16.7/19.9
Ba	201.9	705.9	506.1 ± 76.14	None	4	201.9/467.9/537.7/678.8
Br	1.1	49.8	15.3 ± 9.39	Square root	3	4.6/13/27.1
Ca	1851	19982	6517 ± 3086.4	Log + 1	3	3975/6191/11341
Cd	0	0.96	0.25 ± 0.3	Square root	3	0.03/0.42/0.75
Cl	226.6	479.3	319.1 ± 51.61	Log + 1	4	247/285/330/398
Cs	1.9	8.6	5.2 ± 1.57	None	5	2.6/4.4/5.5/6.7/7.7
Cu	12.4	37.8	30.2 ± 4.44	None	6	12.4/23.6/27.9/30.9/33.4/37.5
Fe	14401	72385	40405 ± 9518.21	None	5	18560/30703/40135/46277/72385
Ge	0	0.7	0.07 ± 0.18	Square root	2	0.01/0.54
I	0	9.6	2.21 ± 2.46	Log + 1	2	0.12/4.5
In	0	1.4	0.54 ± 0.48	Log + 1	4	0.01/0.43/0.81/1.18
La	19.7	66.9	46.25 ± 7.98	None	5	26/41/46/54/65
Mn	142.9	815.3	410.3 ± 151.1	Log + 1	5	202/298/370/510/765
Mo	4.2	12.4	6.19 ± 1.5	Log + 1	4	4.7/5.7/7.3/12.4
Nb	12.4	20.8	18.3 ± 1.36	None	4	16/17.9/18.6/19.5
Nd	61.7	188.0	105 ± 24.17	Log + 1	4	77.1/99.4/117.6/158.2
Pb	11.1	22.9	17.91 ± 2.34	None	7	11.1/14.8/15.9/16.6/17.8/19.5/21.4
S	0	691.2	40.68 ± 125.35	Log + 1	2	10.7/435.1
Sb	0	1.8	0.23 ± 0.49	Square root	2	0.01/0.15
Si	7142	37273	29778 ± 5757.3	None	5	7142/20792/27045/31776/35451
Sn	0.8	4.1	2.58 ± 0.76	None	5	1.01/1.90/2.55/2.92/3.58
Sr	82.2	269.1	184.8 ± 24.81	None	3	82.2/183.4/262.9
Ta	0	10.8	1.76 ± 2.68	Log + 1	5	0.13/2.62/4.85/6.85/10.87
Te	0	1.5	0.31 ± 0.47	Square root	2	0.03/1.02
Th	6.6	15.7	11.22 ± 1.75	None	2	9.85/12.5
Tl	0	0.9	0.06 ± 0.19	Square root	2	0.01/0.63
Y	11.9	32.1	22.44 ± 2.83	None	6	11.9/18.9/21.8/23.1/24.9/30
Zn	71.6	481.8	123.64 ± 60.43	Log + 1	4	91.9/119.9/171.1/481.8
Zr	94	284.6	221.32 ± 33.57	None	6	94/177/204/221/237/267
